# Determination of reference ranges for normal upper trapezius elasticity during different shoulder abduction using shear wave elastography: a preliminary study

**DOI:** 10.1038/s41598-020-74307-2

**Published:** 2020-10-13

**Authors:** Lei Wang, Xi Xiang, Bi-Hui Zhu, Li Qiu

**Affiliations:** 1 Department of Ultrasound, West China Hospital of Sichuan University, No. 37 Guo Xue Xiang, Chengdu, 610041 Sichuan Province China; 2 Department of Ultrasound, Sichuan Academy of Medical Sciences & Sichuan Provincial People’s Hospital, Chinese Academy of Sciences Sichuan Translational Medicine Research Hospital, Chengdu, 610072 Sichuan Province China

**Keywords:** Medical imaging, Ultrasonography, Orthopaedics, Fatigue, Pain, Musculoskeletal system, Muscle, Anatomy, Health care, Health occupations, Signs and symptoms

## Abstract

The purpose of this study was to determine the reference ranges of normal upper trapezius (UT) elasticity during different shoulder abduction using shear wave elastography (SWE). Mean shear wave velocity (SWV) of UT elasticity in eighty healthy participants were measured at left and right shoulder 0° abduction and 90° passive abduction (L0°, R0°, L90°, R90°) with SWE. The effects of potential factors (gender, UT thickness, age, and body mass index) on UT elasticity were analyzed. The reference ranges of normal UT elasticity were calculated by using the normal distribution method. UT elasticity was significantly different among various shoulder abduction (P < 0.0001). UT elasticity was significantly higher in males at both L90° (P < 0.05) and R90° (P < 0.01) than in females. The reference ranges of normal UT elasticity were 2.90–4.01 m/s at L0° and 3.01–4.29 m/s at R0°, and were 4.90–6.40 m/s in males and 4.40–6.20 m/s in females at L90°, 5.20–7.02 m/s in males and 4.71–6.80 m/s in females at R90°. Our results suggest that gender should be considered when determining the reference ranges of normal UT elasticity at L90° and R90° with SWE. These values may provide quantitative baseline measurements for the assessment of UT muscle strain in the future.

## Introduction

Neck pain is a significant health problem, ranking as the fourth greatest contributor to global disability^1^. Some evidence suggests that occupation (e.g., office computer workers, professional drivers, medical workers, etc.) and sedentary working postures (e.g., poor mouse or keyboard position) may be related to the onset of neck pain^2–4^. With the change of lifestyle, growing people use computers or electronic products to work and maintain sedentary sitting position for long periods, which can easily induce overstretch of neck extensor muscles. Increased tension in the extensor muscles of the neck mainly caused by passive overstretch can lead to muscle fatigue or pain^5–8^. Upper trapezius (UT) is a large and superficial extensor muscle of the neck, which is prone to muscle strain due to passive overstretch. The initial symptoms of UT muscle strain mostly manifest as poor sense of walking stability, dizziness and fatigue; Muscle fatigue can be considered an objective biomarker of cumulative exposure due to repetitive activities or postures^8,9^. Early UT muscle strain which may be characterized by muscle fatigue is hard to be diagnosed by conventional methods. These methods have some limitations: clinical assessment cannot quantify muscle function, isokinetic strength testing can show only synergistic muscle rather than an individual muscle force^10^, and an individual muscle activity assessed by electromyography (EMG) may be influenced by electrical activity in adjacent muscles^11–13^. Moreover, UT muscle strain is easy to be misdiagnosed or even delayed treatment, which may lead to serious consequences such as disability. However, changes in the UT elasticity can be used to estimate tension changes of the UT responding to passive overstretch^5,7,13^. In addition, the UT elasticity which indirectly represents muscle contractile function may change in the early stage of muscle strain^13^. Therefore, it is imperative to find new means for evaluating the UT elasticity.

Fortunately, new methods such as strain elastography, acoustic radiation force impulse (ARFI), and shear wave elastography (SWE, such as supersonic shear imaging) allow the assessment of the UT elasticity^14–16^. Compared with EMG, SWE has an advantage in measuring low muscle activation levels^9,12,13^, such as the UT in the early muscle strain. Compared with ARFI and strain elastography, SWE has the advantages of good reproducibility and satisfactory reliability^17^. Alfuraih et al.^18^ showed that SWE can quantitatively assess muscle elasticity and provide a noninvasive, real-time, and reproducible measurement with few operator dependencies. UT elasticity can be easily measured due to its parallel consistent muscle fibers; The measurement results are reliable and reproducible by aligning the long axis of the transducer with the muscle fiber orientation^17,19^. Because there exists a strong linear relationship between muscle elasticity and passive tension when muscles are passively overstretched^5,13^, UT muscle strain can be assessed by SWE. Furthermore, during shoulder abduction, previous study demonstrated excellent intra- and inter-operator reproducibility for the UT elasticity^20,25^.

Here we used shear wave velocity (SWV) measured by SWE as an index of the UT elasticity. Because the original measurement recorded with SWE is SWV, which is then mathematically converted to Young’s modulus for each pixel. In addition, there are additional potential inaccuracies associated with converting SWV to Young’s modulus. Hence, for measurements of the UT elasticity, SWV can potentially be more reliable than Young’s modulus^17,21^.

More importantly, if the reference range of the UT elasticity is established in healthy individuals using SWE, it may be helpful to further assess UT muscle strain. Nevertheless, the assessment for normal reference range of the UT elasticity has not been tested yet. Moreover, there are almost no studies on the reference ranges of normal UT elasticity during different shoulder abduction.

Thus, to evaluate early UT muscle strain, a reference range of the UT elasticity in healthy individuals assessed by SWE is required. The aim of this study was to determine the reference ranges of normal UT elasticity at shoulder 0° and 90° abduction, which may provide a preliminary quantitative baseline measurements reference for assessment of the UT muscle strain.

## Methods

### Participants

From December 2018 to May 2019, 80 healthy individuals (35 males, 45 females) were consecutively recruited. The inclusion criteria were as follows: (i) no pain in the neck and shoulder over the last 2 years, (ii) no fatigue and weakness in the neck muscles over the last 6 months, (iii) no regular sports or fitness training during the last year. The exclusion criteria were as follows: (i) musculoskeletal diseases, (ii) a history of metabolic disorders, rheumatic or endocrine diseases, carcinoma, surgical procedures in the region of the chest, neck and shoulder girdle, neck trauma and neurological dysfunctions, (iii) patients who are taking myorelaxants and other drugs affecting UT elasticity, (iv) Pregnant women. Informed consent for the acquisition and analysis of imaging data was obtained from the participants before starting their examinations. Besides, this study was approved by the West China Hospital of Sichuan University Ethics Committee and performed in accordance with the Declaration of Helsinki.

### SWE ultrasound examinations

**Figure 1:**
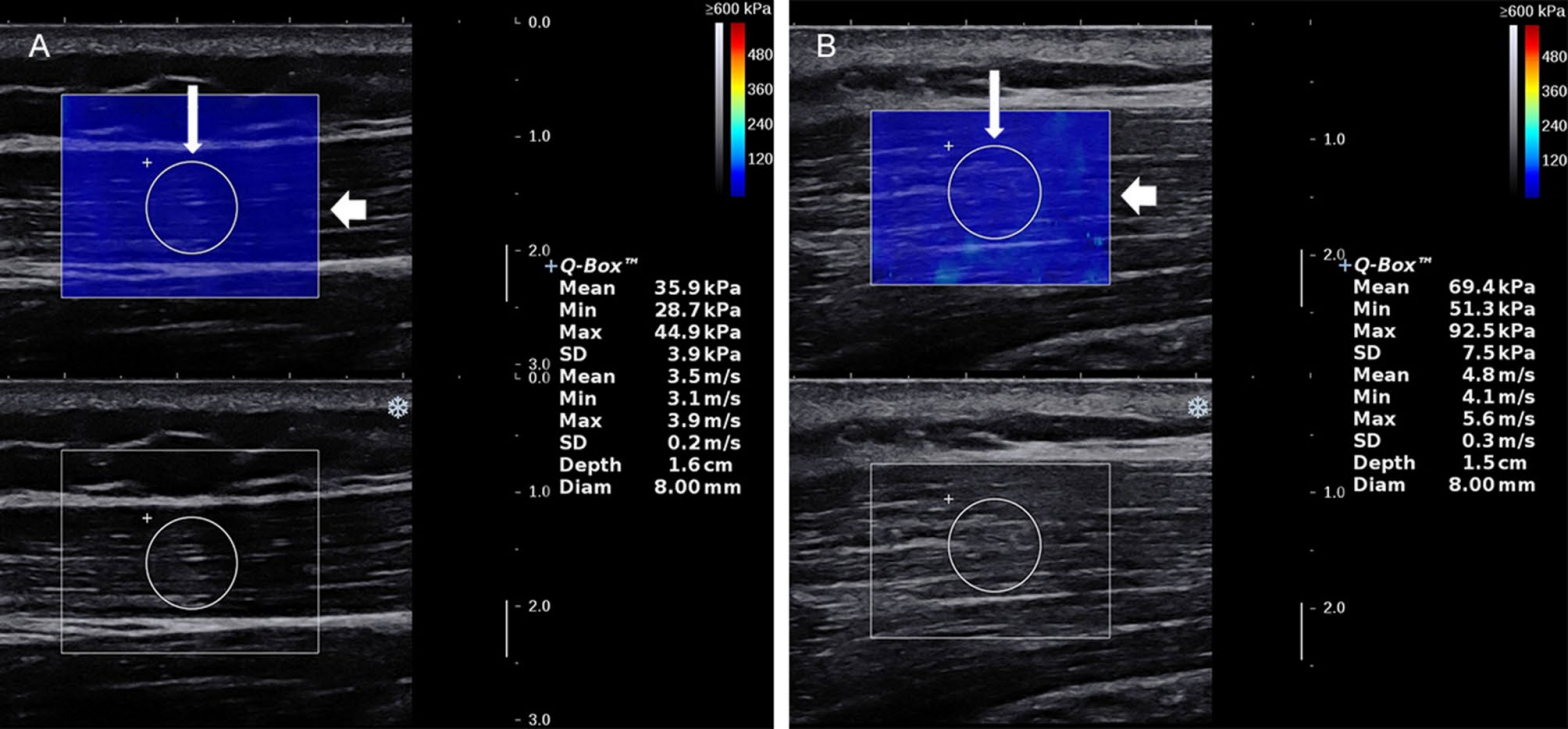
SWE-mode (top panel) and B-mode (bottom panel) ultrasound images of the UT. A color map of UT elasticity is shown in the square region of interest (ROI) (thick arrow). The mean SWV in the round Q-box (thin arrow) is presented on the right side of the image. **A**,**B** show that the size of the Q-box is 8 mm in diameter. The mean SWV of a healthy volunteer at R0° was 3.5 m/s (**A**), the mean SWV at R90° was 4.8 m/s (**B**).

The device used to obtain B-mode and SWE images of the UT was Aixplorer US system (SuperSonic Imagine, Aix-en-Provence, France), with an SL 15–4 linear probe operating at 4–15 MHz. US device parameters were as follows: the superficial musculoskeletal preset in the standard default mode, smoothing level 5, persistence high and 0–600 kPa (scale). The tip of transducer covered with several millimeters of ultrasound gel was placed smoothly to the targeted area of UT along perpendicular direction to the skin^22,23^. It was of importance to ensure that there was no pressure between skin and the probe. The operator maintained the transducer for a few seconds to obtain a stable SWE image. The size of the square region of interest (ROI) was adjusted according to UT thickness. ROI depth varied between 0.5 cm and 2.5 cm from the skin because the targeted UT muscle fibers are consistently parallel, superficial, and ROI colormap was stable and even in this study. Mean SWV was measured in a round area called Q-box: the size of Q-box was fixed as a diameter of 8 mm, Q-box positioning was at the center of the ROI, avoiding the deep and superficial fascia of the UT. Shoulder abduction positions for bilateral examination of each participant were as follows: (i) shoulder 0° abduction (L0° and R0°), (ii) shoulder 90° passive abduction (L90° and R90°). To avoid the influence of scapular depression and shoulder joint rotation on the UT elasticity^24^, all the participants were strictly examined according to the following specific procedure: (i) During shoulder 0° abduction, the participants sat and stayed fully relax, and upper arm rested gently against the chest wall (0° abduction angle), with upper arm in the same plane of the chest wall, forearm in pronation, forearm and hand resting flat on top of hip and thigh; (ii) During shoulder 90° passive abduction, arm was passively positioned at shoulder 90° abduction on a plinth supported with the arm in the same plane of the chest wall, with the elbow fully extended and the thumb pointed to the ceiling (performed in the frontal plane at 90° abduction angle). The angle of shoulder abduction was measured by a manual goniometer to ensure the reliability and consistency. Figure 1 illustrates that the system automatically calculated the SWV values for the Q-box area expressed in m/s at R0° and R90°.

### Study protocol

The participants were prohibited from exercising for 48 h before the experiment. Before testing, the participants were asked to have a 5 min rest in a seated position. The examining time of all the participants were fixed between 9:00 a.m. and 12:00 a.m. The ultrasound examination was performed on all the participants by two experienced sonographers (A and B) who received SWE training. They were blinded to each other’s results for the entire study. All participants were examined by operator A. Furthermore, 20 participants were selected randomly for consistency analysis. These 20 participants were examined by operator B and then measured again by operator A on the second day. Participants were asked to maintain each abduction position for thirty seconds to cooperate with our inspection and obtain stable SWE images, and then were asked to relax for 1 min between each measurement to avoid muscle fatigue. All the acquisition and measurements were carried out with the longitudinal axis of the probe parallel to the long axis of muscle fibers to obtain good reliability and repeatability^17,19,25^. The measurement site of the target UT was marked at the midpoint between the seventh cervical spinous process and the acromion^20,25^. The mean SWV in SWE images was measured three times in each shoulder abduction position by the same operator, averaged, and expressed in m/s.

### Statistical analysis

The data were analyzed with SPSS 20.0 software (IBM, Armonk, NY, USA); P < 0.05 was the two-side significance level. The Kolmogorov–Smirnov test was used to assess the normality of continuous variables. The intraclass correlation coefficient (ICC) and standard error of measurement (SEM) were calculated, and goodness of fit (R^2^) was obtained by linear regression analysis to determine the intra- and inter-operator reproducibility. A two-way ANOVA (within-subject factors: shoulder side and shoulder abduction angle) was used to test the interaction between these two within-subject factors. Independent sample t-test for a binary variable (gender), Paired t-test between different shoulder abduction, and Pearson correlation test for continuous variables (UT thickness, age, BMI) were used. The lower and upper limits of the reference ranges of normal UT elasticity were mean SWV − 1.96 × SD and mean SWV + 1.96 × SD, respectively, where SD is the standard deviation of mean SWV.

## Results

### Study population

Table 1 shows basic characteristics of 80 healthy individuals. There was a total of 35 males and 45 females. They are all right-handed. The mean age ± SD was 42.80 ± 11.40 years (range 19–70 years). The mean BMI ± SD was 23.60 ± 2.70 kg/m^2^ (range 16–32 kg/m^2^). The mean UT thickness ± SD was 10.00 ± 1.01 mm at L0°, 10.60 ± 1.18 mm at R0°, 13.60 ± 1.20 mm at L90°, 14.50 ± 1.21 mm at R90°, respectively.

**Table 1:** Basic characteristics of the 80 healthy volunteers in this study.

Characteristics	Healthy volunteers
Number	80
**Age (y)**	
Mean ± SD (Range)	42.80 ± 11.40 (19–70)
Gender: Female/Male	45/35
BMI (kg/m^2^)	23.60 ± 2.70
**UT thickness (mm)**	
L0°	10.00 ± 1.01
R0°	10.60 ± 1.18
L90°	13.60 ± 1.20
R90°	14.50 ± 1.21

Data are presented as mean ± SD (range).

*SD* Standard deviation, *BMI* body mass index, *UT* upper trapezius, *mm* millimeter, *L0°* left shoulder 0° abduction, *R0°* right shoulder 0° abduction, *L90°* left shoulder 90° passive abduction, *R90°* right shoulder 90° passive abduction.

### Reliability analysis

The intra- and inter-operator reproducibility of the mean shear wave velocity of the UT at left and right shoulder 0° and 90° abduction are listed in Tables 2 and 3. The ICC values show the intra-operator reproducibility at L0° (ICC = 0.84), R0° (ICC = 0.95), L90° (ICC = 0.97), and R90° (ICC = 0.98); and the inter-operator reproducibility at L0° (ICC = 0.89), R0° (ICC = 0.92), L90° (ICC = 0.96), and R90° (ICC = 0.97).

**Table 2:** Intra-operator reproducibility of shear wave velocity at different shoulder abduction.

Shoulder abduction	SWV, m/s		ICC	SEM	P	R^2^
	Operator A	Operator Aʹ				
L0°	3.48 ± 0.28*	3.32 ± 0.56	0.84	0.08	< 0.01	0.654
R0°	3.66 ± 0.32	3.59 ± 0.29	0.95	0.04	< 0.01	0.929
L90°	5.58 ± 0.41	5.53 ± 0.72	0.97	0.03	< 0.01	0.932
R90°	5.93 ± 0.49	5.91 ± 0.66	0.98	0.02	< 0.01	0.971

*ICC* intra-class correlation coefficient, *SWV* mean shear wave velocity, *SEM* standard error of measurement, *R*^*2*^ goodness of fit.

*Mean** ± **standard deviation.

**Table 3:** Inter-operator reproducibility of shear wave velocity at different shoulder abduction.

Shoulder abduction	SWV, m/s		ICC	SEM	P	R^2^
	Operator A	Operator B				
L0°	3.48 ± 0.28*	3.60 ± 0.39	0.89	0.07	< 0.01	0.755
R0°	3.66 ± 0.32	3.57 ± 0.78	0.92	0.05	< 0.01	0.862
L90°	5.58 ± 0.41	5.52 ± 0.61	0.96	0.04	< 0.01	0.902
R90°	5.93 ± 0.49	5.90 ± 0.52	0.97	0.03	< 0.01	0.965

*ICC* intra-class correlation coefficient, *SWV* mean shear wave velocity, *SEM* standard error of measurement, *R*^*2*^ goodness of fit.

*Mean** ± **standard deviation.

### Influence of different shoulder abduction

**Figure 2:**
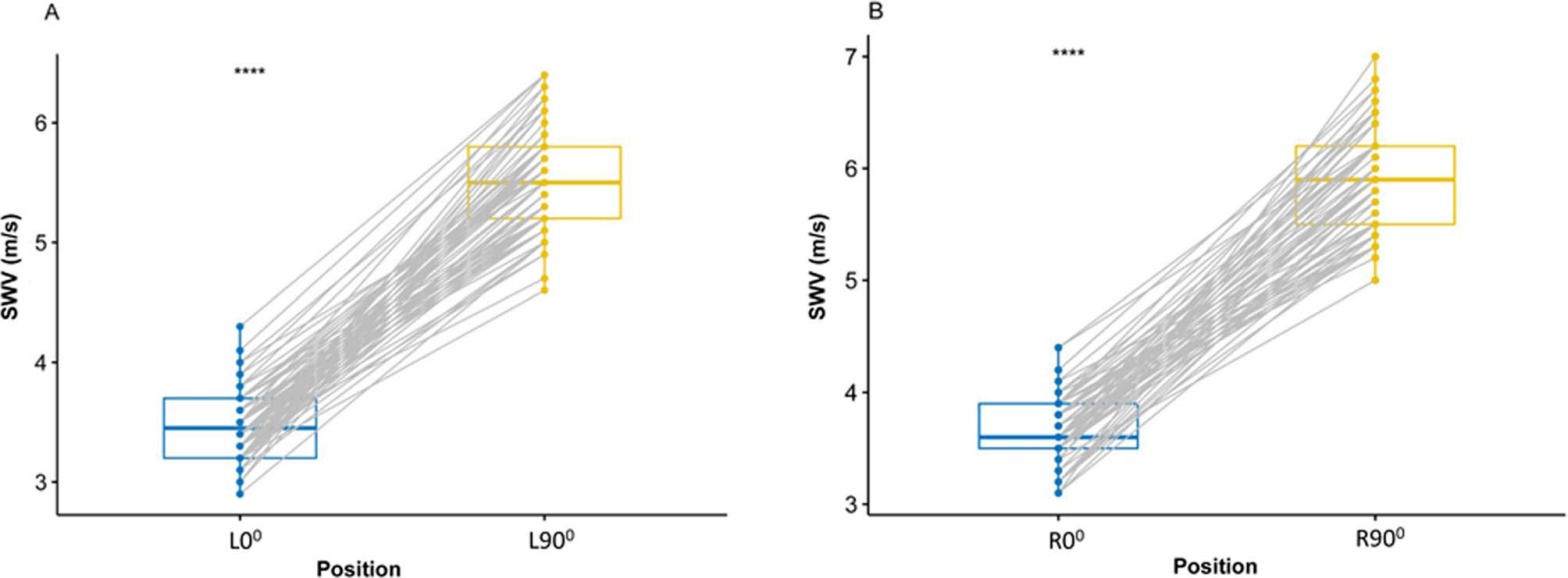
Box-and-whisker plots of the SWV in healthy volunteers with SWE between L0° and L90° (**A**), between R0° and R90° (**B**) through the paired t test. The top and bottom of each box represent the 75th and 25th percentiles, respectively; the median, the minimum, and maximum are represented by the horizontal line, as well as the top and bottom of the whiskers, respectively. All pairs of compared groups showed a significant difference. ****P < 0.0001, *SWV* mean shear wave velocity.

**Figure 3:**
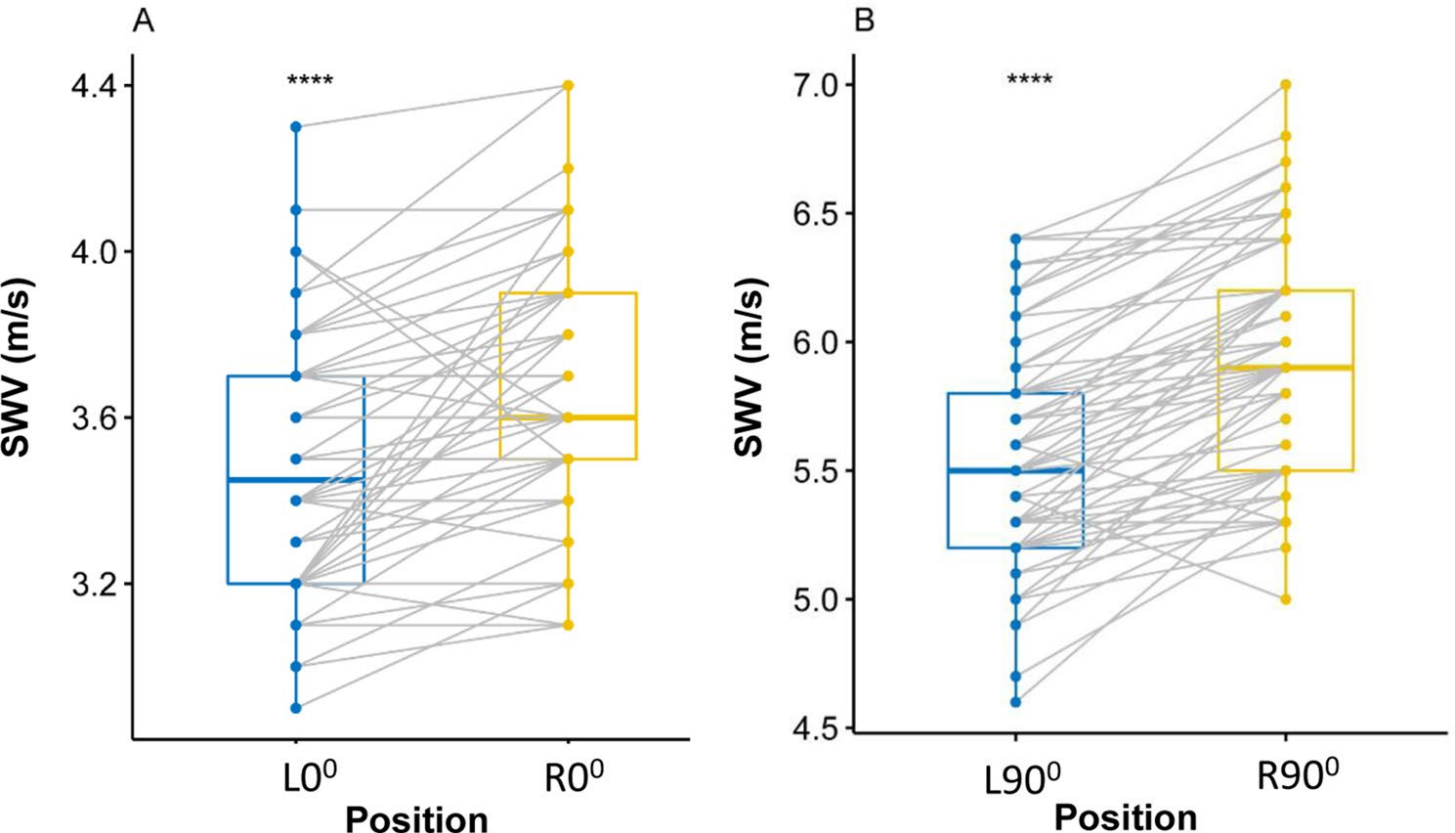
Box-and-whisker plots of the SWV in healthy volunteers with SWE between L0° and R0° (**A**), between L90° and R90° (**B**) through the paired t test. The top and bottom of each box represent the 75th and 25th percentiles, respectively; the median, the minimum, and maximum are represented by the horizontal line, as well as the top and bottom of the whiskers, respectively. All pairs of compared groups showed a significant difference. ****P < 0.0001, *SWV* mean shear wave velocity.

No interaction between shoulder side and shoulder abduction angle was found (F = 3.5; P = 0.062) (Table 4). Mean SWV was statistically significantly higher at L90° than L0° (P < 0.0001) (Fig. 2A), higher at R90° than R0° (P < 0.0001) (Fig. 2B), higher at R0° than L0° (P < 0.0001) (Fig. 3A), and higher at R90° than L90° (P < 0.0001) (Fig. 3B). The mean SWV was 3.48 m/s at L0°, 3.66 m/s at R0°, 5.54 m/s at L90°, and 5.88 m/s at R90°, respectively.

**Table 4:** Results of two-way ANOVA with within-subject factors ‘shoulder side’ and ‘shoulder abduction angle’.

Within-subject factor	F	P
Shoulder side	37.49	< 0.001
Shoulder abduction angle	2.37	< 0.001
Side × angle interaction	3.50	0.062

Side × angle interaction = interaction between shoulder side and shoulder abduction angle.

### Influence of gender

**Figure 4:**
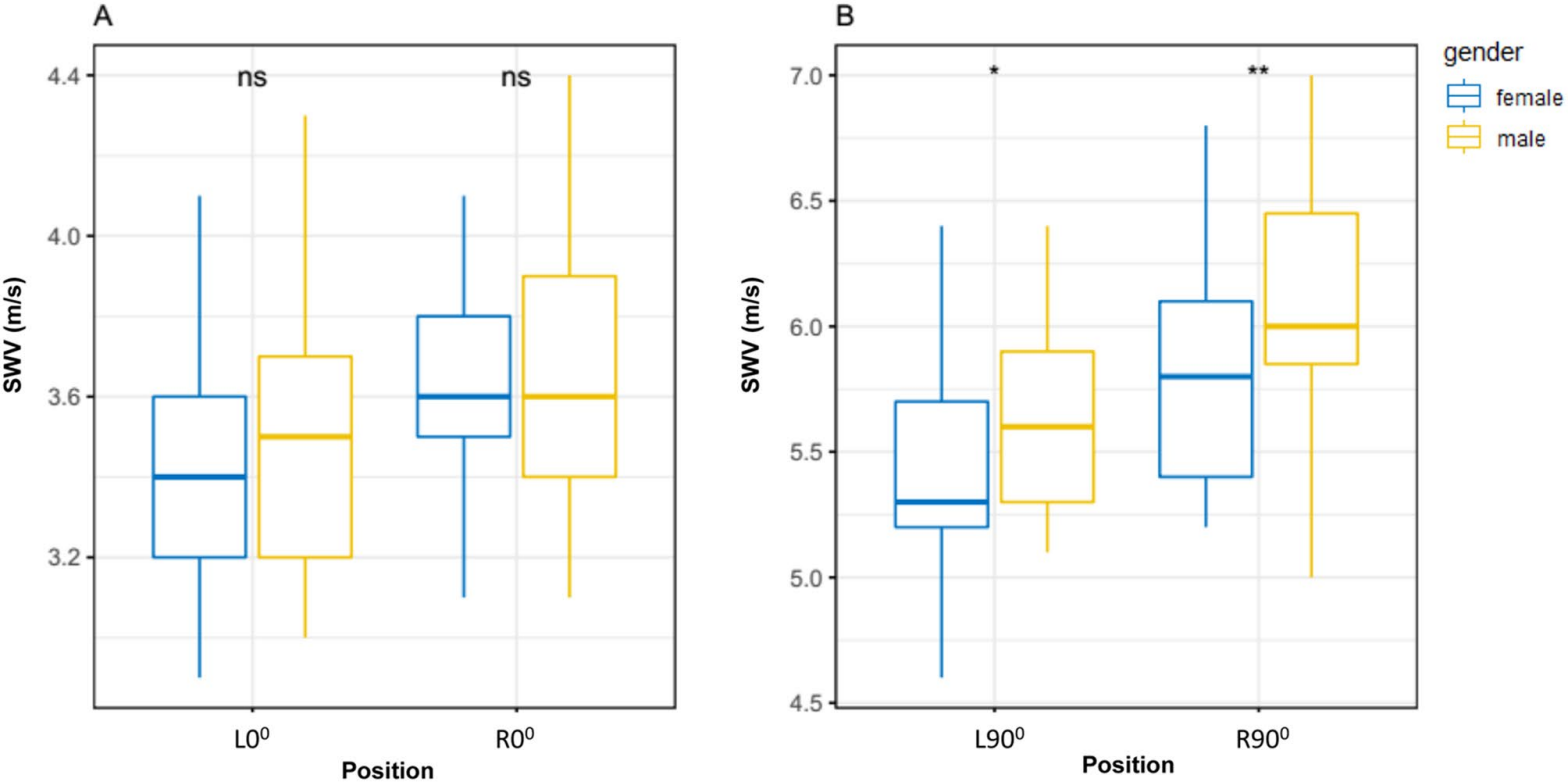
Box-and-whisker plots of the SWV in healthy volunteers between males and females at both L0° and R0° (**A**), both L90° and R90° (**B**). The top and bottom of each box represent the 75th and 25th percentiles, respectively; the median, the minimum, and maximum are represented by the horizontal line, as well as the top and bottom of the whiskers, respectively. All pairs of compared groups showed a significant difference except that no statistical significance was found in SWV between males and females at both L0° and R0°, *P < 0.05, **P < 0.01, ns = no statistically significance; SWV = mean shear wave velocity.

Mean SWV at both L0° (P = 0.56, > 0.05) and R0° (P = 0.61, > 0.05) were not statistically significantly different between females and males (Fig. 4A), but mean SWV at both L90° (P < 0.05) and R90° (P < 0.01) were statistically significantly higher in males than in females (Fig. 4B).

### Influence of UT thickness

**Figure 5:**
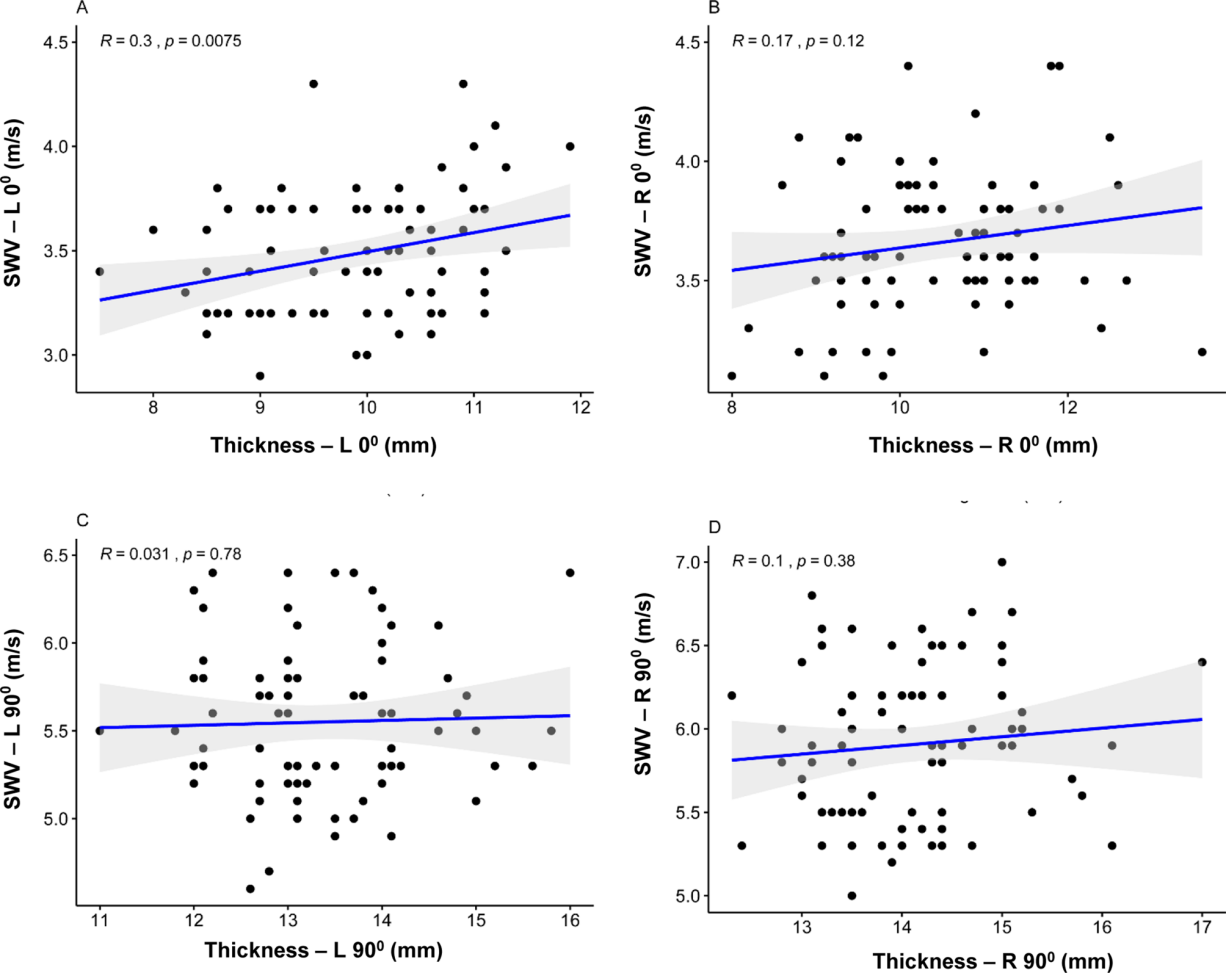
Scatterplots of the SWV and UT thickness in healthy volunteers at L0° (**A**), R0° (**B**), L90° (**C**), and R90° (**D**). *SWV* mean shear wave velocity, *UT* upper trapezius.

UT thickness was not statistically significantly correlated with mean SWV at R0° (R = 0.17, P = 0.12, > 0.05), L90° (R = 0.03, P = 0.78, > 0.05) and R90° (R = 0.10, P = 0.38, > 0.05) (Fig. 5B–D). Slight statistically positive correlation can be ignored at L0° (R = 0.30, P < 0.01) (Fig. 5A) between mean SWV and UT thickness.

### Influence of age

**Figure 6:**
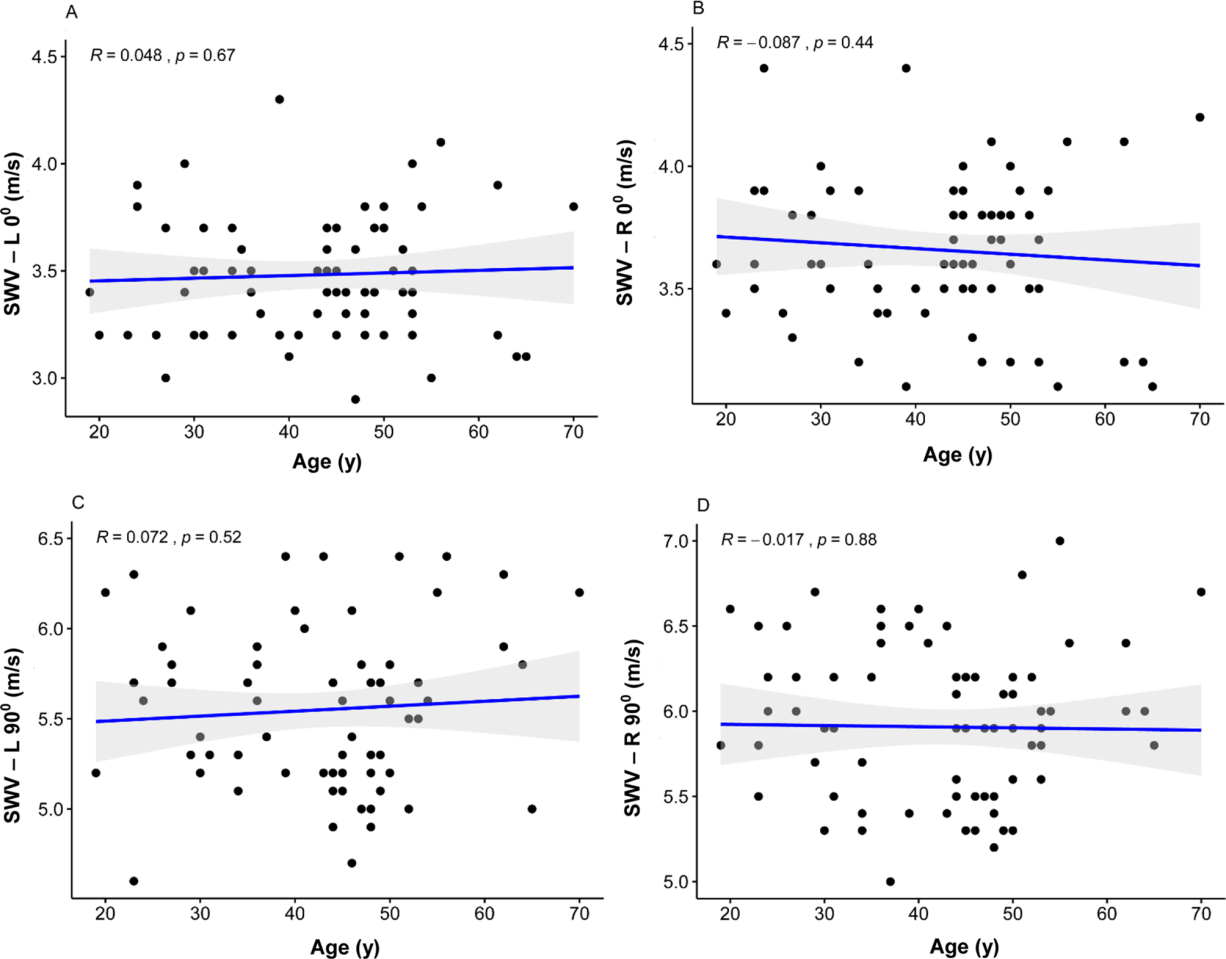
Scatterplots of the SWV and age in healthy volunteers at L0° (**A**), R0° (**B**), L90° (**C**), and R90° (**D**). *SWV* mean shear wave velocity.

There was no statistically significant correlation between age and mean SWV at L0° (R = 0.05, P = 0.67, > 0.05), R0° (R = 0.09, P = 0.44, > 0.05), L90° (R = 0.07, P = 0.52, > 0.05) and R90° (R = 0.02, P = 0.88, > 0.05) (Fig. 6A–D).

### Influence of BMI

**Figure 7:**
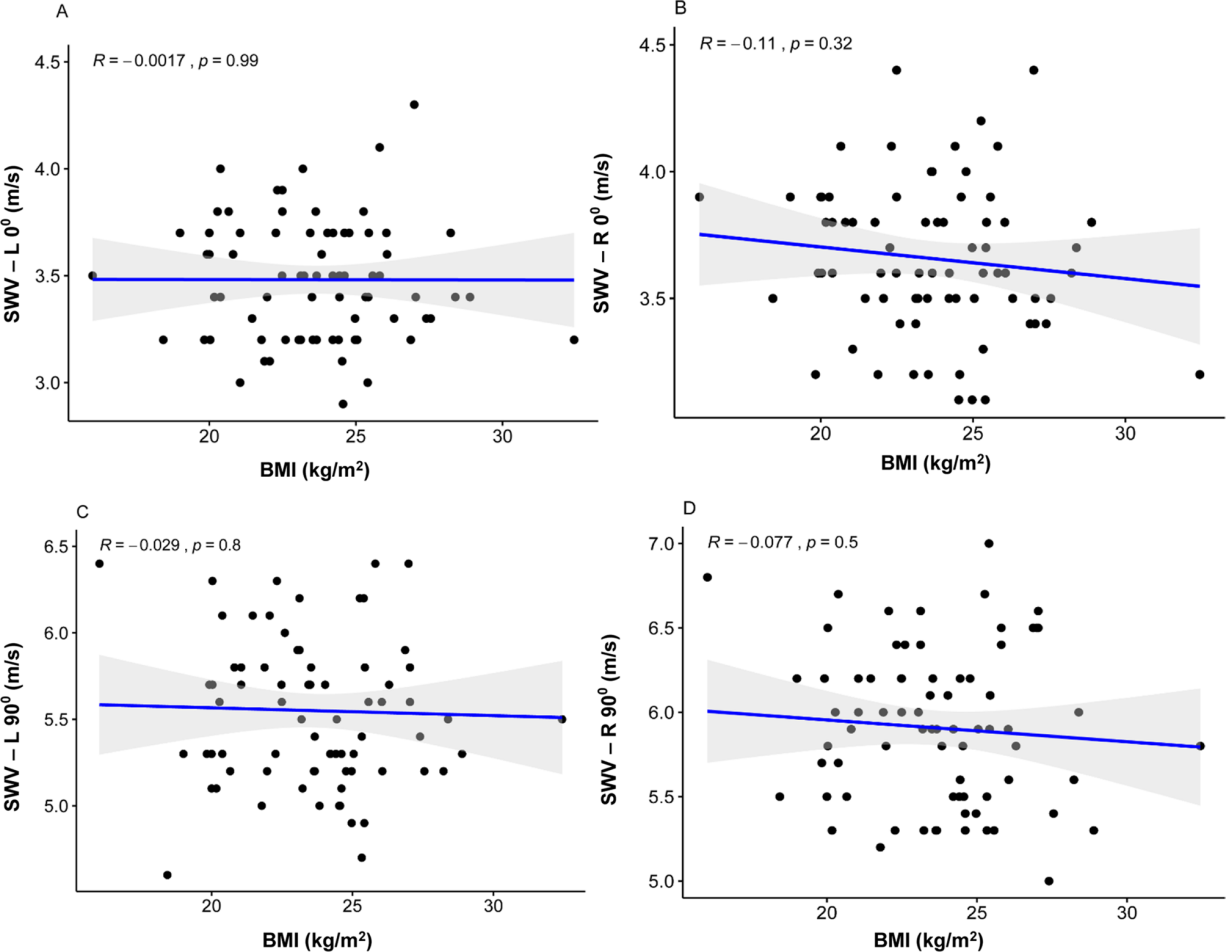
Scatterplots of the SWV and BMI in healthy volunteers at L0° (**A**), R0° (**B**), L90° (**C**), and R90° (**D**). *SWV* mean shear wave velocity, *BMI* body mass index.

The BMI was not statistically significantly correlated with mean SWV at L0° (R = − 0.001, P = 0.99, > 0.05), R0° (R = − 0.11, P = 0.32, > 0.05), L90° (R = − 0.03, P = 0.80, > 0.05) and R90° (R = − 0.08, P = 0.50, > 0.05) (Fig. 7A–D).

### Reference ranges setup

According to the above analyses, the reference ranges of normal UT elasticity at L90° and R90° were affected by gender, yet UT elasticity at L0° and R0° could be utilized for combined reference ranges regardless of gender, UT thickness, age, and BMI. The reference ranges of normal UT elasticity were 2.90–4.01 m/s at L0° and 3.01–4.29 m/s at R0° for both males and females, and were 4.90–6.40 m/s in males and 4.40–6.20 m/s in females at L90°, 5.20–7.02 m/s in males and 4.71–6.80 m/s in females at R90° (Table 5).

**Table 5:** The reference ranges of the upper trapezius elasticity in 80 healthy volunteers during different shoulder abduction.

Shoulder abduction	Gender	SWV, m/s		
		Mean ± SD	95% CI	Reference range
L0°	Male/female	3.48 ± 0.28	3.42–3.55	2.90–4.01
R0°	Male/female	3.66 ± 0.32	3.59–3.74	3.01–4.29
L90°	Male	5.68 ± 0.38	5.56–5.80	4.90–6.40
	Female	5.43 ± 0.51	5.28–5.58	4.40–6.20
R90°	Male	6.07 ± 0.46	5.92–6.22	5.20–7.02
	Female	5.73 ± 0.55	5.56–5.89	4.71–6.80

*SWV* mean shear wave velocity, *SD* standard deviation, *CI* confidence interval, *L0°* left shoulder 0° abduction, *R0°* right shoulder 0° abduction, *L90°* left shoulder 90° passive abduction, *R90°* right shoulder 90° passive abduction.

## Discussion

Taking advantage of the SWE, the present study found that UT elasticity was significantly higher on the right than on the left at both shoulder 0° abduction (P < 0.0001) and 90° abduction (P < 0.0001). It may be because right arm is used more frequently than left one (all the participants were right-handed in this study). Moreover, UT elasticity was significantly higher at L90° than L0° (P < 0.0001), at R90° than R0° (P < 0.0001). It may be due to increased muscle tension and contractility of the UT during shoulder 90° abduction. Particularly, gender had impact on UT elasticity at both L90° (P < 0.05) and R90° (P < 0.01) in the present study, maybe indicating there is a significant difference in force-generating and contractile capacity of the UT muscle between genders during shoulder 90° abduction^26–28^. A possible explanation for this discrepancy may be the obvious difference in muscle size and composition between genders at shoulder 90° abduction^29,30^. Haizlip et al.^31^ showed that collagen and elastin architectures of muscle fibers mainly determined UT elasticity, which may vary between different gender. Change in muscle elasticity can indirectly reflect changes of muscle mass and quality^27,30^. Besides, changes in muscle fiber type and cross-section area of neck muscles in patients with neck or shoulder pain have been reported in previous literature^26,31,32^.

In the present study, sTable 2 and 3 showed that the results of these parameters (including ICC, SEM, R^2^) reflected the excellent intra- and inter- operator reproducibility of the UT elasticity measurement during different abduction. We found excellent intra-operator reproducibility in using SWE to measure the elasticity of the UT at L0° (ICC = 0.84), R0° (ICC = 0.95), L90° (ICC = 0.97) and R90° (ICC = 0.98) done by the same operator. This shows that SWE is a reliable technique for assessing UT elasticity. Our results revealed excellent inter-operator reproducibility for the measurements of the UT elasticity at L0° (ICC = 0.89), R0° (ICC = 0.92), L90° (ICC = 0.96) and R90° (ICC = 0.97). The reasons for such high intra- and inter-operator reproducibility in our study are as follows: standardized measuring method, advanced ultrasound device, standardized training and skilled operators, and the muscle fibers of the target UT are consistently parallel, superficial (UT elasticity is less affected by the depth), and SWE color images are stable and even. Our findings are similar with the previous study showing excellent reliability in measuring UT elasticity using SWE at shoulder 0° and 30° abduction^20^.

Several unique features of the present study should be pointed out. Firstly, to our knowledge, this was the first study to quantitatively assess UT elasticity during four different shoulder abduction positions. Reviewing the literature on the neck extensor muscle elasticity using SWE, we found that most researchers performed muscle elasticity in different head postures^33–35^, few studies have focused on muscle elasticity during different shoulder abduction. Additionally, most studies on muscle elasticity using SWE are about the Young's modulus, the SWV of muscle elasticity has been rarely studied. For the UT elasticity measurements, because measurements of the SWV may provide better reliability of study results, we recommend SWV as a surrogate parameter for UT elasticity instead of Young’s modulus^17,21^. Secondly, this work may be the first study to evaluate potential factors affecting UT elasticity with SWE, including gender, age, UT thickness and BMI. No previous studies have analyzed so many factors on muscle elasticity as we did^36,37^. Thirdly and most important, the clinical significance of this study lies in providing quantitative baseline measurements for UT muscle strain through determining the reference ranges of normal UT elasticity during different shoulder abduction with SWE. Because the early diagnosis of the UT muscle strain is difficult, these reference ranges may help to judge whether someone’s UT elasticity is within the ranges of normal values^13,38–40^. We determined the reference ranges of normal UT elasticity in different shoulder abduction positions that may indicate the different contractile functions, which has not been studied yet. In addition, both shoulder 0° and 90° abduction positions are easy to be operated with strict consistency, so our research results are highly reliable and feasible. This method may be extended to the quantitative evaluation reference for early UT muscle strain using SWE in the future multicenter study.

There are several limitations in the current research. The number of participants investigated is still relatively small. Moreover, because this is a preliminary study, the effects of confounding factors, such as age, might have been underestimated. Inclusion of a larger study population with wider age and BMI ranges would be crucial to validate these results and might alter the significance of such confounding factors on UT elasticity. These aspects need to be further refined in the future.

In conclusion, the current research suggest that gender should be considered when determining the reference ranges of normal UT elasticity at L90° and R90° with SWE. These values may provide a preliminary quantitative baseline measurements reference for the assessment of UT muscle strain. In the future research, we will attempt to increase sample size in identifying the reference ranges of normal UT elasticity during different shoulder abduction.

## Data Availability

The datasets generated during and/or analysed during the current study are available from the corresponding author on reasonable request.
